# Highlighting the Biological Potential of the Brown Seaweed *Fucus spiralis* for Skin Applications

**DOI:** 10.3390/antiox9070611

**Published:** 2020-07-11

**Authors:** Rafaela Freitas, Alice Martins, Joana Silva, Celso Alves, Susete Pinteus, Joana Alves, Fernando Teodoro, Helena Margarida Ribeiro, Lídia Gonçalves, Željko Petrovski, Luís Branco, Rui Pedrosa

**Affiliations:** 1 MARE—Marine and Environmental Sciences Centre, ESTM, Politécnico de Leiria, 2520-641 Peniche, Portugal; rafaelapatriciafreirefreitas@gmail.com (R.F.); joana.m.silva@ipleiria.pt (J.S.); celso.alves@ipleiria.pt (C.A.); susete.pinteus@ipleiria.pt (S.P.); joana.abr.alves@gmail.com (J.A.); fjtdgm@gmail.com (F.T.); 2 Research Institute for Medicines (iMed.ULisboa), Faculty of Pharmacy, Universidade de Lisboa, Avenida Professor Gama Pinto, 1649-038 Lisboa, Portugal; hribeiro@campus.ul.pt (H.M.R.); lgoncalves@ff.ulisboa.pt (L.G.); 3 Departamento de Química, REQUIMTE-CQFB, Faculdade de Ciências e Tecnologia da Universidade Nova de Lisboa, 2829-516 Caparica, Portugal; z.petrovski@fct.unl.pt (Ž.P.); l.branco@fct.unl.pt (L.B.)

**Keywords:** *Fucus spiralis*, seaweeds, antioxidant, oxidative stress, photoprotective, anti-enzymatic, anti-inflammatory, skin microbiome, dermo-cosmetics

## Abstract

Skin aging is a biological process influenced by intrinsic and extrinsic factors. The last ones, mainly exposure to UV radiation, increases reactive oxygen species (ROS) production leading to a loss of extracellular matrix, also enhanced by enzymatic degradation of matrix supporting molecules. Thus, and with the growing demand for eco-friendly skin products, natural compounds extracted from brown seaweeds revealed to be good candidates due to their broad range of bioactivities, especially as antioxidants. The aim of this study was to assess the dermo-cosmetic potential of different fractions obtained from the brown seaweed *Fucus spiralis*. For this purpose, in vitro antioxidant (Total Phenolic Content (TPC), 1,1-Diphenyl-2-picrylhydrazyl (DPPH) radical scavenging activity, Ferric Reducing Antioxidant Power (FRAP), Oxygen Radical Absorbance Capacity (ORAC)), anti-enzymatic (collagenase, elastase and hyaluronidase), antimicrobial, anti-inflammatory (NO production) and photoprotective (ROS production) capacities were evaluated. Although nearly all fractions evidenced antioxidant effects, fraction F10 demonstrated the highest antioxidant ability (EC_50_ of 38.5 µg/mL, DPPH assay), and exhibited a strong effect as an inhibitor of collagenase (0.037 µg/mL) and elastase (3.0 µg/mL). Moreover, this fraction was also the most potent on reducing ROS production promoted by H_2_O_2_ (IC_50_ of 41.3 µg/mL) and by UVB (IC_50_ of 31.3 µg/mL). These bioactivities can be attributed to its high content of phlorotannins, as evaluated by LC-MS analysis, reinforcing the potential of *F. spiralis* for further dermatological applications.

## 1. Introduction

Skin is the mirror of health and beauty. In modern societies, skin is also related with status, and thus there is a massive demand for efficient products that can delay the signs of skin aging. The cosmetic industry represents one of the most profitable and rapidly growing business area in the world economy, essentially due to the consumers’ increasing demand for new products, with both protective and therapeutic benefits. This kind of skincare products can mediate distinct well-being and health effects such as anti-aging, anti-acne, photoprotective, wound healing, and skin whitening [1,2].

Skin aging is a complex phenomenon involving intrinsic and extrinsic factors. While intrinsic factors affect all organs in the same way as skin, the latter is the result of skin exposure to external factors, such as pollution and ultra violet (UV) radiation. On both processes, reactive oxygen species (ROS) play a key role [3,4,5,6]. Although the human body is equipped with an efficient defense machinery, physical stressors (pollution, ultra violet light, etc.), internal factors (age, diseases, etc.), and an unhealthy lifestyle (poor quality diet, alcohol, smoking, etc.) can contribute to a redox instability, unbalancing the antioxidant defense mechanisms and favoring ROS production, leading to an oxidative stress condition. This condition is believed to be directly related to aging phenomena. Comprising three layers, the epidermis, dermis, and a deeper subcutaneous tissue, skin is mainly composed by keratin, and is the first line of defense of the human body against physical stressors, acting as a shield [4,7]. UV light is a potent promoter of ROS, which trigger several metabolic pathways leading to the overexpression of enzymes responsible for collagen degradation, such as matrix metalloproteinases (MMP-1, MMP-3 and MMP-9), thus inhibiting the expression of procollagen-1. Skin aging is also associated with loss of skin moisture, in which hyaluronic acid (HA) plays an important role. This molecule is a glycosaminoglycan that, due to its high polarity, can retain water molecules, maintaining skin hydration, being also involved in skin repair metabolism and protection against UV damage [8]. Continuous exposure to UV light will increase ROS levels which, in turn, promote elastase, collagenase and hyaluronidase production, consequently leading to the degradation of elastin, collagen, and hyaluronic acid, resulting in wrinkles, thinner skin, potential damage, and sagging [7]. A healthy life style including a diet rich in antioxidants and vitamins [4,6], coupled together with proper skin care, is believed to decrease oxidative stress associated with the development of aging signs.

Cosmetic industries are continuously searching for new products, with special focus on antioxidant molecules, specially from natural origin to answer the increasing consumers’ demand for “natural,” “green,” and “eco-friendly” bioactive solutions, as efficient alternatives to chemically orchestrated additives, such as synthetic preservatives [9,10]. From this perspective, nature has revealed to be a great source of new bioactive ingredients for incorporation into innovative formulations, presenting several advantages such as fewer side effects, safer use and environment friendliness [11]. Moreover, these natural components were previously described to promote distinct functional activities of interest for skincare products development, exhibiting great ability to target several key players linked to ageing and skin degradation, such as matrix metalloproteinases, oxidative damage and inflammatory events [1,4].

In the last decades, marine organisms, including seaweeds, have been targeted as potential sources of new active ingredients, distinct from the ones derived from terrestrial environments, with multiple health benefits [12,13,14,15,16]. Seaweeds have in their constitution several functional components, such as essential amino acids and proteins, carbohydrates, pigments, minerals, polysaccharides, dietary fibers, vitamins, polyunsaturated fatty acids, phenolics, and bioactive secondary metabolites, that have great potential to be used in innovative skincare formulations [10,17,18]. Among seaweeds, several reports have attested the high nutritional and functional value of the genus *Fucus* which has been described as an excellent source of dietary fibers and minerals, with a high array of bioactive compounds such as fucoidan, phlorotannins, fucoxanthin, among others, possessing numerous biological activities including antioxidant, anti-inflammatory, antitumor, antimicrobial, and others [18]. Regarding skin applications, most studies were conducted with *Fucus vesiculosus*, which revealed several cosmetic properties, including stimulation of collagen production and expression of heme oxygenase-1 molecule, antioxidant, anti-inflammatory, inhibitory effects on enzymes related to skin aging and degradation, and increased expression of integrin molecules [19,20]. Moreover, the potential of its extracts was also evaluated by in vitro studies, showing capacity to protect against UV radiation, reduce wrinkle depth, increase skin brightening and decrease skin thickness. However, the evidences related to the dermo-cosmetic potential of *Fucus spiralis* extracts and/or compounds are scarce. Accordingly, the main goal of this work was to study the potential of a set of extracts from *Fucus spiralis* through the evaluation of their in vitro antioxidant, anti-inflammatory, antimicrobial, anti-enzymatic, and photoprotective properties to be applied in skin care products.

## 2. Materials and Methods

### 2.1. Solvents and Reagents

Solvents of analytical and HPLC grades were purchased from VWR-BDH Chemicals (Fontenay-sous-Bois, France), Fischer Scientific (Loughborough, UK), and Honeywell Riedel-de-Haën (Illkirch, France) while ultrapure water was obtained from an Advantage A10 Milli-Q lab equipment (Merck, Darmstadt, Germany). Analytical grade reagents from different suppliers were used to perform the in vitro bioassays, e.g., antioxidant capacity: Merck (Darmstadt, Germany), Sigma-Aldrich (Steinheim, Germany), and AlfaAesar (Karlsruhe, Germany); antimicrobial activities: VWR-BDH Chemicals-Prolabo (Leuven, Belgium); antienzymatic assays: Sigma-Aldrich (Steinheim, Germany), Invitrogen-ThermoFisher Scientific (Waltham, MA, USA); anti-inflammatory properties: Merck (Darmstadt, Germany), Sigma-Aldrich, (St. Louis, MO, USA), Lonza (Basel, Switzerland), and Biowest (Riverside, MO, USA). Reagents and culture media for in vitro cellular assays were supplied by Merck (Darmstadt, Germany), Gibco (Grand Island, NY, USA), Invitrogen (Life Technologies, Warrington, UK), and Sigma (Seelze, Germany).

### 2.2. Seaweed Collection and Preparation

The brown seaweed *Fucus spiralis* (Linnaeus, 1753) was harvested in October 2018 in Consolação beach, Peniche, Portugal (39°19′30.4″ N 9°21′36.8″ W), and was identified by Dr. Susete Pinteus, a marine biologist with vast experience in seaweed identification. After cleaned from debris and epiphytes, *F. spiralis* was frozen at −20 °C and freeze-dried (Scanvac Cool Safe, LaboGene, Lynge, Denmark). Dehydrated material (ref: FS201810) was then ground into a powder in a grinder which was stored protected from light, at room temperature.

### 2.3. Seaweed Extraction and Fractionation

**Figure 1:**
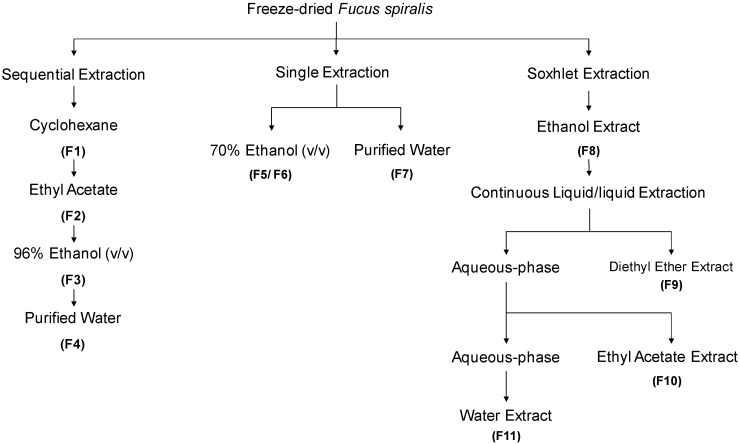
Overview of *Fucus spiralis* samples’ extraction and fractionation methodologies.

The extraction/fractionation process of *Fucus spiralis* biomass is depicted in Figure 1. Four extraction methodologies were outlined, with three of them being performed at room temperature, and one by using a Soxhlet extractor, affording eleven fractions (F1-F11). The powdered seaweed material (50 g) was extracted with different solvents (4 × 200 mL), at constant stirring, for 4 h, in the dark. The first extraction was performed sequentially with cyclohexane, ethyl acetate, ethanol and water, resulting in four fractions (F1–F4). In parallel, single extractions were also accomplished, one with ethanol: water (70/30, *v*/*v*, 200 mL) affording two fractions, a less polar (F5) and a more polar one (F6), and another one, only with water (200 mL), affording a single fraction (F7). For the Soxhlet extraction, a solution of ethanol:water (70/30, *v*/*v*, 200 mL) was used. The resulting extract was evaporated until dryness giving a complex fraction (F8).Then, this fraction was re-suspended in hot (80 °C) water (200 mL), cooled, and subjected to a L/L partition, firstly with diethyl ether (3 × 100 mL), and then with ethyl acetate (5 × 100 mL). Organic phases were dried with anhydrous Na_2_SO_4_, filtered, and concentrated to dryness resulting into the diethyl ether (F9), ethyl acetate (F10) and aqueous (F11) fractions. All the extracts were filtered with qualitative filter paper nr. 4 (VWR International, Alfragide, Portugal) and concentrated under reduced pressure, at low temperature (40 °C), in a rotary evaporator (IKA HB10) and/or in a speed-vacuum equipment (Eppendorf Concentrator Plus, Leicestershire, UK), while aqueous extracts were freeze-dried (Scanvac Cool Safe, LaboGene, Lynge, Denmark).

### 2.4. Evaluation of Fucus spiralis Biological Properties

For in vitro bioassays, samples and controls were dissolved in water and/or dimethyl sulfoxide (DMSO).

#### 2.4.1. Quantification of Total Phenolic Content (TPC)

The total phenolic content was estimated based on the procedure developed by Singleton and Rossi (1965) [21] adapted to microscale [22] which is based on the colorimetric reaction of phenolic substances with Folin-Ciocalteu reagent. The results were extrapolated from a standard curve obtained with phloroglucinol and thus expressed as mg of phloroglucinol equivalents per gram of dry extract (mg PE/g of extract).

#### 2.4.2. Antioxidant Capacity

The antioxidant capacity was analyzed through three different, well established, biochemical methods, namely 1,1-diphenyl-2-picrylhydrazyl (DPPH) radical scavenging activity, ferric reducing antioxidant power (FRAP), and oxygen radical absorbance capacity (ORAC), as follows:

##### 1,1-Diphenyl-2-picrylhydrazyl (DPPH) Radical Scavenging Activity

The ability of *F. spiralis* samples to scavenge the DPPH radical was conducted as previously described by Brand-Williams and co-workers [23]. For all the samples with capacity to scavenge DPPH radical in more than 50%, the EC_50_ was determined.

##### Oxygen Radical Absorbance Capacity (ORAC)

The ORAC assay was performed as described by Dávalos and co-workers [24]. Seaweed fractions (20 µL) were pre-incubated with fluorescein (120 µL; 70 nmol/L) for 15 min at 37 °C. After that, a solution of 2,2′-Azobis(2-methylpropionamidine) dihydrochloride (AAPH) (60 µL; 12 mmol/L) was added and the fluorescence (*λ* excitation: 458 nm; *λ* emission: 520 nm) was recorded every min for 4 h. Trolox was used as standard antioxidant and the results were expressed as µmol of Trolox equivalents per g of dry extract (µmol TE/g of extract).

##### Ferric Reducing Antioxidant Power (FRAP)

The FRAP method was performed according to Benzie and Strain [25], adapted to microscale with slight adjustments [15]. This method is based on the reduction of Fe^3+^ to Fe^2+^ through the electron donation, which can be mediated by the presence of antioxidant compounds, leading to the formation of an intense blue color proportional to the antioxidant activity. Briefly, FRAP reagent (0.3 mol/L acetate buffer (pH = 3.6), 10 mmol/L TPTZ (2,4,6-Tri(2-pyridyl)-s-triazine) in 40 mmol/L HCl and 20 mmol/L FeCl_3_ at a ratio of 10:1:1) was prepared and incubated at 37 °C. Seaweed fractions (2 µL) were then added to 198 µL FRAP reagent and incubated in the dark for 30 min at 37 °C. After this time, the absorbance increase was measured at 593 nm. FeSO_4_ was used as standard and the results were expressed as µmol/L of FeSO_4_ equivalents per g of dry extract (µmol/L of FeSO_4_ /g of extract).

#### 2.4.3. Enzymatic Activities

The inhibitory effects of *F. spiralis* fractions on collagenase, elastase and hyaluronidase enzymes were evaluated according to the methodologies described below. (−)-Epigallocatechin gallate (EGCG) was used as positive control.

##### Hyaluronidase Activity

Hyaluronidase activity was evaluated according to Yahaya and Don [26], adapted to microscale. Briefly, hyaluronic acid (HA) forms a turbid suspension when an acid solution is added, while in the presence of hyaluronidase (7 U/mL), HA depolymerization occurs and turbidity is decreased. The turbidity is directly proportional to the amount of HA [27]. Thus, the inhibitory ability of seaweed fractions was measured by the amount of HA remaining in the mixed solution. A 20 mmol/L sodium phosphate with 77 mmol/L sodium chloride and 0.01% bovine serum albumin (BSA) solution was used to prepare the enzyme diluent (pH 7.0, 37 °C) and a 24 mmol/L sodium acetate with 79 mmol/L acetic acid and a 0.1% BSA solution was used to prepare an acidic albumin solution (pH 3.75, 25 °C). Briefly, 3 μL of each sample was mixed with 5 μL of hyaluronidase and 67 μL of enzyme diluent, and pre-incubated for 10 min at 37 °C. After the pre-incubation time, the reaction was initiated with the addition of 25 μL of HA (0.03% in 300 mmol/L sodium phosphate, pH 5.35, 37 °C) and incubated for 45 min at 37 °C. HA was then precipitated using 200 μL of acidic albumin, the mixture was left at room temperature for 10 min and the absorbance was measured at 600 nm. A blank of each sample was also performed (3 μL of sample, 97 μL of enzyme diluent and 200 μL of acidic albumin). The absorbance in the absence of enzyme was used as control value for maximum inhibition. The percentage of hyaluronic acid was calculated as: hyaluronic acid (% of control) = ((Abs_sample_ − Abs_sample blank_)/Abs_control_)) × 100, where Abs_sample_ is the absorbance of sample with enzyme and HA, Abs_sample blank_ is the absorbance of the sample in acidic albumin solution without enzyme and HA, and Abs_control_ is the absorbance of the HA without the presence of the enzyme or samples.

##### Collagenase Activity

The anti-collagenase activity was determined using the EnzChek™ Gelatinase/Collagenase Assay Kit (# E12055, Invitrogen™, ThermoFisher Scientific) according to manufacturer’s instructions. Collagenase activity was calculated through the slope of the linear phase of the fluorescence resulting from the cleavage of fluorescein-conjugate gelatin mediated by the enzyme. The fluorescence of produced peptides is proportional to the proteolytic activity. The results were expressed as arbitrary fluorescence units per minute (∆ fluorescence (a.u.)/min) as percentage of the control.

##### Elastase Activity

The anti-elastase activity was determined using the EnzChek™ Elastase Assay Kit (# E12056, Invitrogen™, ThermoFisher Scientific) according to manufacturer’s instructions. Elastase activity was calculated through the slope of the linear phase of the fluorescence resulting from the cleavage of the conjugate supplied in the kit. The results were expressed as arbitrary fluorescence units per minute (∆ fluorescence (a.u.)/min) as percentage of the control.

#### 2.4.4. Antimicrobial Activity

Antimicrobial activity of seaweed fractions was evaluated against two Gram (+) bacteria, *Staphylococcus epidermidis* (DSM 1798) and *Cutibacterium acnes* (DSM 1897), and one fungus, *Malassezia furfur* (DSM 6170), previously acquired from the German Collection of Microorganisms and Cell Cultures (DSMZ) biobank. For each microorganism the following growth conditions and media were used: Trypticase Soy Broth at 37 °C (*S. epidermidis*), Tryptic Soy Broth with anaerobic conditions at 37 °C (*C. acnes*), and Leeming-Notman medium at 30 °C (*M*. *furfur*). The antimicrobial activity was accompanied by optical density at 600 nm in order to verify the ability of fractions (1000 µg/mL) to inhibit microorganisms’ growth in the exponential phase. Results were expressed as percentage of growth inhibition relative to the control (growth medium with microorganism).

#### 2.4.5. Evaluation of Biological Activities of *F. spiralis* Fractions on In Vitro Cellular Models

The cytotoxic, anti-inflammatory, cytoprotective and photoprotective activities of *F. spiralis* fractions were evaluated on different cellular models. Details of each methodology are described below.

##### Maintenance of Cell Culture

RAW 264.7 (ATCC-TIB-71), 3T3 (ACC-173) and HaCaT (300493) cells were acquired from the American Type Culture Collection (ATCC), German Collection of Microorganisms and Cell Cultures (DSMZ), and Cell Lines Services Germany (CLS) biobanks, respectively. The RAW 264.7 and 3T3 cells were cultured in Dulbecco’s Modified Eagle’s medium: Nutrient Mix F-12 (DMEM/F-12) supplemented with 10% fetal bovine serum (FBS), 100 IU/mL penicillin, and 100 μg/mL streptomycin. The RAW 264.7 culture medium was also supplemented with 1% sodium pyruvate. The HaCaT cells were cultured in RPMI 1640 medium supplemented with 10% FBS, 100 IU of penicillin G (sodium salt) and 100 μg of streptomycin sulfate, and 2 mmol/L L-glutamine. Cells were kept in a 95% moisture and 5% CO_2_ at 37 °C. Subculture was performed according to biobank instructions whenever cultures reached 80–85% confluence.

##### Evaluation of Cytotoxicity

The cytotoxic activities of seaweed extracts were evaluated on RAW264.7 (5 × 10^4^ cells/well), 3T3 (1.5 × 10^4^ cells/well) and HaCaT cells (4 × 10^4^ cells/well) after seeding in 96-well plates and incubated overnight. Cells were then treated with the aforementioned fractions (1000 µg/mL) for 24 h. Untreated cells were used as control. Saponin (Sigma, 47036-50G-F) was used as a cellular death positive control (100% of cell death). After that, the effects were estimated using the 3-[4–dimethylthiazol-2-yl]-2, 5-diphenyltetrazolium bromide (MTT) colorimetric assay, as described by Mosmann [28].The intracellular formazan crystals were then extracted and solubilized with dimethyl sulfoxide (DMSO) and the absorbance was measured at 570 nm using a microplate reader (Synergy H1 Multi-Mode Microplate Reader, BioTek^®^ Instruments, Vermont, VT, USA). The results were expressed as percentage of control untreated cells.

##### Quantification of Nitric Oxide (NO) on RAW 264.7 Cells

The inflammatory and anti-inflammatory effects of *F. spiralis* fractions were estimated through the NO production according to Yang and co-workers [29] with slight modifications. RAW 264.7 cells were seeded in 96-well plates at a density of 5 × 10^4^ cells/well, and incubated for 16 h before the assay. The cells were then treated with seaweed fractions for 24 h using sub-toxic concentrations to evaluate their ability to increase NO levels. On the other hand, lipopolysaccharides (LPS) at 1 μg/mL were used to induce an inflammatory condition in order to evaluate the ability of fractions to fight the inflammatory state. Accordingly, the cells were pre-treated with fractions for 1 h before the LPS addition and incubated for 24 h. After that, 100 μL of culture medium from each well were transferred to a new plate, and 100 μL of Griess reagent (1% (*w*/*v*) sulfanilamide, 0.1% (*w*/*v*) N-(1-naphthyl) ethylenediamine in 2.5% (*v*/*v*) phosphoric acid) were added. Dexamethasone was used as positive control. The plate was then incubated in the dark, at room temperature, for 30 min, and the absorbance was measured at 546 nm. Results were expressed as percentage of control untreated cells.

##### Reactive Oxygen Species (ROS) Production on HaCaT Cells

The levels of ROS were determined after the submission of HaCaT cells to two different oxidative stress conditions, one induced by hydrogen peroxide (500 μmol/L) and another induced by exposure to UV radiation. The ability of seaweed extracts to reduce the ROS production was estimated using the fluorescent 2′,7′-dichlorodihydrofluorescein diacetate (H2-DCFDA) probe according to Marto et al. [30]. HaCaT sub-confluent cells were incubated with 20 µM of H2-DCFDA in the dark, for 30 min at 37 °C. The cells’ medium was then changed for fresh medium and cells were treated with the samples (1000 µg/mL) and/or controls for 1 h. Regarding the hydrogen peroxide (H_2_O_2_) assay, cells were treated simultaneously with H_2_O_2_ (500 µmol/L) and fractions. H_2_O_2_ was used as a positive control for ROS production. Concerning UV assay, three UVB lamps (Sankyo Denki G8T5E, Kanagawa, Japan) with a peak emission at 312 nm were used as UVB source, which was measured using a VLX 312 radiometer equipped with a UVB sensor (Vilber Lourmat, Marne-la-Vallée France). The cells were irradiated with a UVB single dose of 26 mJ/cm^2^ for 15 min in the presence of seaweed fractions (1000 µg/mL) and controls. Ascorbic acid was used as a positive control. The ROS levels were estimated at wavelengths of 485 nm (excitation) and 520 nm (emission) using a fluorescence microplate reader (Synergy H1 Multi-Mode Microplate Reader, BioTek^®^ Instruments, Vermont, VT, USA). Results were expressed as percentage of ROS reduction.

#### 2.4.6. Chemical Characterization

*F. spiralis* fractions were analyzed by UV-Vis spectroscopy, while a deeper chemical characterization of the most promising fraction was performed through HPLC-DAD and LC-MS analysis.

##### UV–Visible Absorption Spectra

Samples were dissolved in dichloromethane, methanol, or methanol/water, and their UV-visible absorption spectra, covering a range of 200–800 nm, was determined using a UV-Visible spectrophotometer Evolution 201, from Thermo Scientific (Madison, WI, USA).

##### LC-MS Analysis

The HPLC system comprised a liquid chromatograph equipped with a Thermo Surveyor pump and an Accela UV/Vis detector (Thermo Scientific, Waltham, MA, USA). Separation was performed on a Nova-Pak C18 column (150 mm length × 3.9 mm i.d., 4 µm particle size, Waters, Milford, MA, USA) thermostated at 30 °C. The mobile phase was composed of eluent A (10% MeOH and 2% CH_3_COOH) and B (90% MeOH and 2% CH_3_COOH). The following elution program was used: 100% A (0–10 min); 85% A and 15% B (10–25 min:); 50% A and 50% B (25–30 min); 30% A and 70% B (50–52 min) and 100% B (52–65 min), flow rate of 0.5 mL/min. The UV detection wavelength was 280 nm.

LC-MS analysis was carried out on a liquid chromatograph LC Agilent 1200 Series coupled to a MS Agilent 6130B Single Quadrupole mass spectrometer (Agilent Technologies, Santa Clara, CA, USA), an apparatus consisting of an autosampler/injector, a column compartment, a binary pump, and an Agilent UV detector with 5 UV channels. The LC separation was conducted with a Poroshell 120 EC-C18 column (410 mm length × 4.6 mm i.d., 2.7 µm particle diameter, Agilent Technologies, Santa Clara, CA, USA) maintained at 30 °C. Elution was performed with a binary solvent system composed of A (0.1% HCOOH in water, *v*/*v*) and B (Acetonitrile) as following: 100% A (0–5 min); 95% A and 5% B (5–30 min); 60% A and 40% B (30–35 min); 10% A and 90% B (35–52 min) and 100% B (52–55 min) at a flow rate 0.3 mL/min. The UV spectral data for all peaks were accumulated with 5 channel UV detector (Agilent q1100 series G1365B MWD serial DE411120174) at 270, 280, 320, and 350 nm, while chromatographic profiles were recorded at 280 nm. Samples were dissolved in water or methanol and all solvents were LC-MS grade. Acquisition of MS data was carried out with the MS Agilent 6130B Single Quadrupole. Nitrogen (99.9% purity) was used and the gas pressure was 206 kPa (30 psi). The instrument was operated in negative and positive ion modes with the ESI needle voltage set at 4.00 kV and temperature of 350 °C. The full scan covered the mass range from *m*/*z* 50 to 1500.

#### 2.4.7. Data and Statistical Analysis

Significant differences between samples and controls were determined using one-way analysis of variance (ANOVA) with Dunnett’s multiple comparison tests. Data which did not meet normal distribution were compared with the Kruskal-Wallis non-parametric test. All data were obtained from at least three independent experiments and are presented as mean ± standard error of the mean (SEM) and considered significant at a level of 0.05 (*p* < 0.05). EC_50_ and IC_50_ were determined using the software GraphPad v5.1 by means of the equation *y* = 100/(1 + 10^(X −logIC50)^). Principal component analysis (PCA) was performed with CANOCO for Windows 4.5 software. Data was also examined with IBM SPSS Statistics 24 (IBM Corporation, Armonk, NY, USA).

## 3. Results

### 3.1. Antioxidant Capacity

The yields of *F. spiralis* fractions (F1-F11), TPC, and their antioxidant capacity assessed through the different assays (DPPH, ORAC and FRAP) are presented in Table 1.

As shown in Table 1, the best extraction yields were achieved with ethanol (F3, F5, F6) and water (F11) fractions, while lower yields were obtained with less polar solvents (F9, F10). Due to the low yield of F9 (0.01%), this fraction was not considered for the outlined in vitro bioassays.

Regarding the total phenolic content (TPC), fraction F10 exhibited the highest value (1679.8 ± 34.0 mg PE/g extract) followed by fractions F3 (362.1 ± 9.7 mg PE/g extract), F5 (309.5 ± 12.7 mg PE/g extract), and F7 (272.0 ± 26.7 mg PE/g extract). Lower amounts of phenolics were detected in fractions F1, F2, F4, F6, and F11. The highest DPPH radical scavenging ability was mediated by F10 which exhibited the smallest EC_50_ value (38.5 µg/mL), followed by F3 fraction (EC_50_ of 157.6 µg/mL), an EC_50_ value very close to those of the standard antioxidant BHT (EC_50_ of 164.5 µg/mL). Similarly, the highest antioxidant activity estimated by ORAC and FRAP assays was also shown by F3, F5, and F10 fractions, while lower antioxidant capacity was denoted by fractions F1 and F11 in these in vitro assays (Table 1).

### 3.2. Enzymatic Activity

The inhibitory effects of seaweed fractions on collagenase, elastase and hyaluronidase activities are displayed in Table 2.

In a first approach, and in the assayed conditions, all the fractions presented some degree of anti-enzymatic activity, at least over one of the tested enzymes. However, the best inhibitory effects were performed by fractions F4, F7, F10, and F11. Fraction F10 exhibited the strongest inhibition over collagenase (IC_50_ of 0.037 µg/mL) followed by F7 (IC_50_ of 4.3 µg/mL), this last one of the same order of magnitude of the standard compound EGCG (IC_50_ of 4.8 µg/mL). A noticeable inhibitory activity over this enzyme was also performed by fractions F11 (IC_50_ of 31.3 µg/mL) and F4 (IC_50_ of 40.4 µg/mL). Regarding the inhibition of elastase, once again, the best results were obtained with F10 (IC_50_ of 3.0 µg/mL), followed by F4 (IC_50_ of 67.8 µg/mL) and F7 (IC_50_ of 123.8 µg/mL) which revealed an inhibitory activity very close to EGCG (IC_50_ of 113.9 µg/mL). The best hyaluronidase inhibitory performance was evidenced by F4 (IC_50_ of 61.1 µg/mL), followed by F2 (IC_50_ of 79.5 µg/mL), and F11 (IC_50_ of 110.1 µg/mL), which also exhibited an inhibitory activity very close to EGCG (IC_50_ of 119.1 µg/mL).

### 3.3. Principal Component Analysis (PCA)

**Figure 2:**
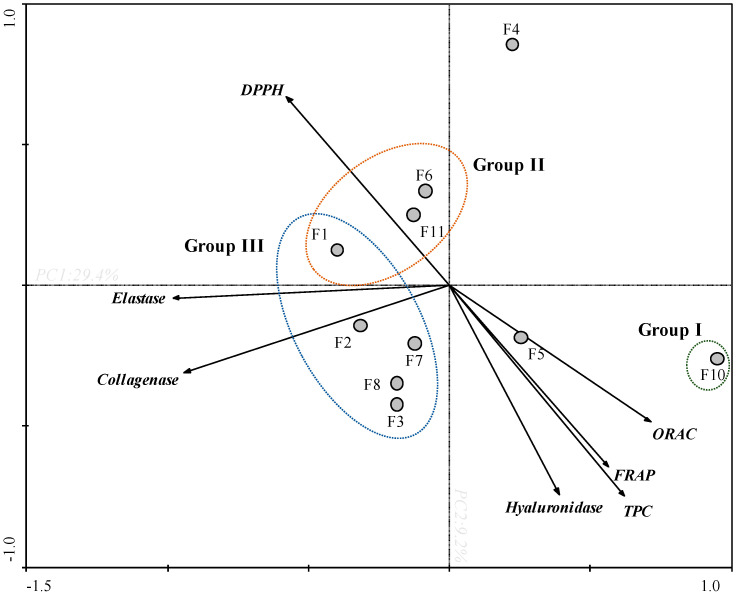
Principal component analysis (PCA) of total phenolic content (TPC), antioxidant activities (DPPH, ORAC and FRAP) and anti-enzymatic activities (collagenase, elastase and hyaluronidase) of *Fucus spiralis* fractions (F1-F11).

In order to obtain an overview of similarities and differences between *F. spiralis* fractions, as well as to establish a relationship between the in vitro bioactivities, a PCA was carried out (Figure 2).

Through the PCA analysis it was possible to observe a clear arrangement between the antioxidant and enzymatic activities (Figure 2). The first two main components explain 29.4% and 9.2% of the total variance of the dataset, respectively. The TPC and antioxidant assays (FRAP and ORAC), and hyaluronidase presented a negative correlation with DPPH, and a null correlation with collagenase and elastase. Among all samples, F10 fraction exhibited the highest antioxidant capacity (Group I) presenting a high TPC content, high values of FRAP and ORAC, and high potential for DPPH scavenging (since the values of this assay were expressed as EC_50_). In the same way, F10 fraction also displayed the highest anti-collagenase and anti-elastase activities exhibiting low IC_50_ values. In turn, F4 fraction has shown high anti-collagenase, anti-elastase, and anti-hyaluronidase activities, which does not appear to be correlated with the antioxidant activity verified by FRAP, DPPH, ORAC and by the TPC. On the other hand, F1, F6 and F11 fractions showed low antioxidant potential (FRAP, TPC, DPPH and ORAC) and low anti-enzymatic activity (collagenase and elastase) (Group II). Within this framework, the fractions present in group III exhibited the lowest potential.

### 3.4. Antimicrobial Activity

**Figure 3:**
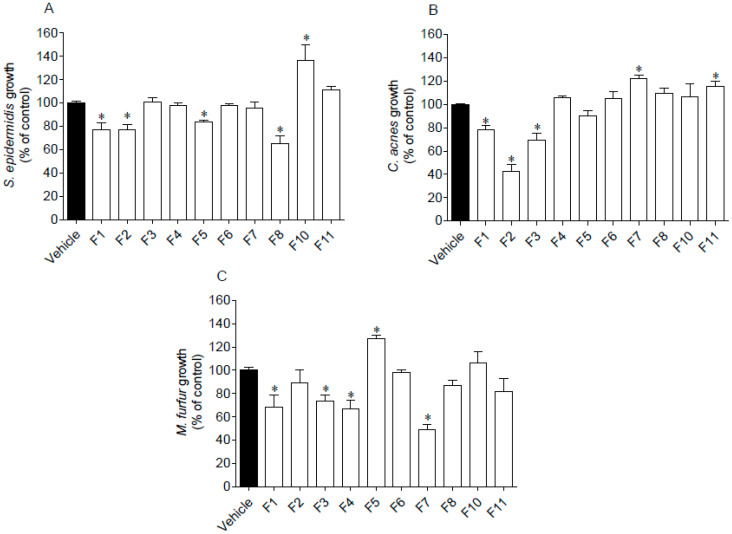
Antimicrobial activity of *Fucus spiralis* fractions (1000 µg/mL) against *Staphylococcus epidermidis* (**A**), *Cutibacterium acnes* (**B**) and *Malassezia furfur* (**C**). Oxytetracycline was used as standard on *C. acnes* and *S. epidermidis* exhibiting an IC_50_ of 0.07 (0.05–0.09) µg/mL and 13.40 (11.22–16.13) µg/mL, respectively. Amphotericin B was used as standard against *M. furfur* growth, revealing an IC_50_ of 11.36 (8.58–14.98) µg/mL. Bars correspond to mean ± SEM of three independent experiments. Symbols (*) represent significant differences (One-way ANOVA, Dunnett’s test; *p* < 0.05) when compared to the vehicle.

The antimicrobial activity of seaweed samples was evaluated against two Gram (+) bacteria, *S. epidermidis* and *C. acnes*, and one fungus, *M. furfur*. Results are presented in Figure 3.

Several seaweed fractions revealed to affect the microorganisms’ growth when tested at 1000 µg/mL (Figure 3). Fraction F1 was the only one that inhibited the growth of the assayed microorganisms. Furthermore, the highest inhibition was mediated by F2 and F7 fractions against *C. acnes* and *M. furfur*, respectively, which reduced the microorganisms’ growth around 50–60%. On the other hand, a stimulation effect was observed on the microorganisms’ growth promoted by fractions F5 (*M. furfur*), F7 and F11 (*C. acnes*), and by F10 (*S. epidermidis*).

### 3.5. Biological Activities of F. spiralis Fractions on In Vitro Cellular Models

#### 3.5.1. Cytotoxicity on 3T3 and HaCaT Cells

**Figure 4:**
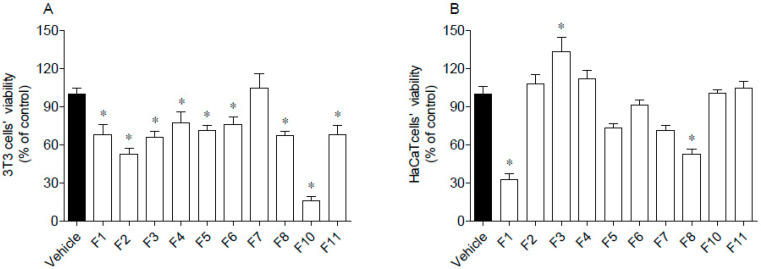
Cytotoxic potential of *Fucus spiralis* samples on 3T3 (**A**) and HaCaT (**B**) cells. Cells’ viability was evaluated after 24 h of exposure to *F. spiralis* fractions (1000 µg/mL) and the results are expressed as % of the control. Bars correspond to mean ± SEM of three independent experiments. Symbols (*) represent significant differences (One-way ANOVA, Dunnett’s test; *p* < 0.05) when compared to the vehicle.

The cytotoxic activities of seaweed samples were evaluated on 3T3 and HaCaT cells, and the results are presented in Figure 4.

Data gathered in Figure 4 suggest that 3T3 cells’ viability was significantly affected by seaweed fractions, except for F7, which did not induce cytotoxicity (Figure 4A). The highest cytotoxicity was mediated by F10 that reduced cells viability by about 80%. Due to the great antioxidant activity of F10, this fraction was subjected to a dose-response analysis (30, 100, 300, 600 µg/mL) and it was verified that at 600 µg/mL, it was no longer toxic. Concerning HaCaT cells, their viability was only affected by exposure to F1 and F8 fractions, which reduced cells’ viability by about 50–70% (Figure 4B). Contrastingly, the F3 fraction stimulated the mitochondrial activity of HaCaT cells.

#### 3.5.2. Nitric Oxide Production on RAW264.7 Cells

**Figure 5:**
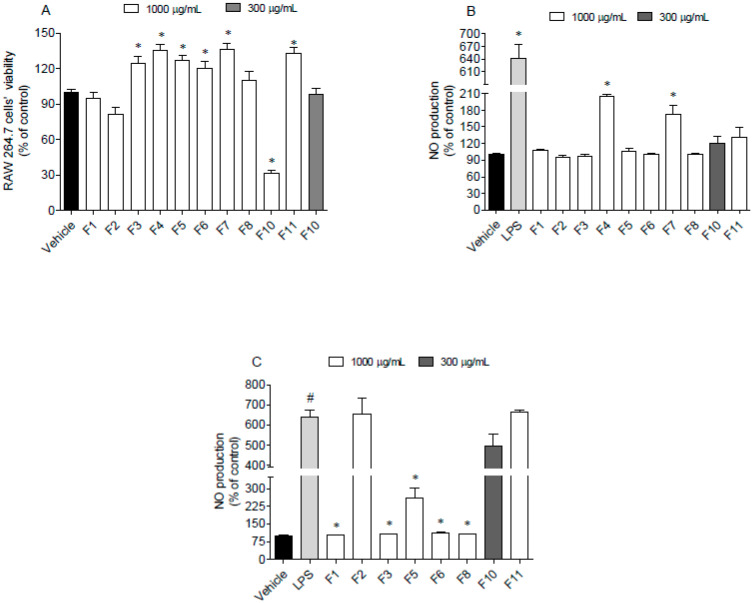
Evaluation of cytotoxicity and anti-inflammatory potential of *Fucus spiralis* samples on RAW 264.7 cells. (**A**) RAW 264.7 cells’ viability following 24 h of exposure to the *F. spiralis* fractions (1000 µg/mL); (**B**) nitric oxide (NO) production by RAW 264.7 cells when exposed to *F. spiralis* fractions (24 h) at sub-toxic concentrations; (**C**) nitric oxide production by LPS-stimulated RAW 264.7 cells in the presence of *F. spiralis* fractions. Bars correspond to mean ± SEM of three independent experiments. Symbols represent significant differences (One-way ANOVA, Dunnett’s test; *p* < 0.05) when compared to: ^#^ vehicle and * LPS.

RAW 264.7 cells were used as a model to address the nitric oxide production induced by lipopolysaccharides (LPS) in the presence and absence of *F. spiralis* fractions. This approach allowed us to understand if fractions induce spontaneous inflammation on cells or if they have ability to protect them in an inflammatory condition when tested at sub-toxic concentrations. The results obtained are shown in Figure 5.

Data gathered in Figure 5 suggest that seaweed fractions at 1000 µg/mL did not induce cytotoxicity on RAW 264.7 cells, excepting for fraction F10, which reduced the cell viability around 70% (Figure 5A). However, when tested at 300 µg/mL, this fraction did not exhibit toxicity. In addition, after the treatment of RAW 264.7 cells with LPS and fractions, it was possible to observe that only F4 and F7 fractions stimulated the NO production (Figure 5B). On the other hand, F1, F3, F5, F6 and F8 fractions decreased the NO levels in the LPS -stimulated RAW 264.7 cells (Figure 5C).

#### 3.5.3. Reactive Oxygen Species (ROS) Production on HaCaT Cells after Exposure to H_2_O_2_ or UVB Light

**Figure 6:**
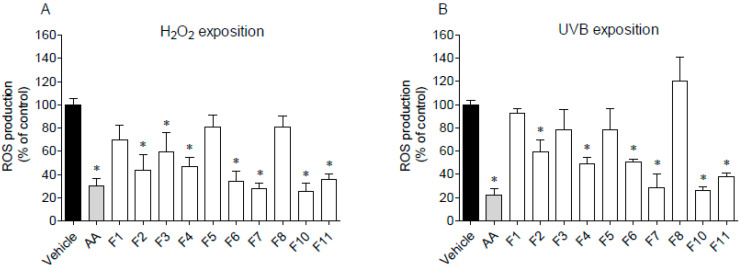
Evaluation of reactive oxygen species (ROS) production by HaCaT cells. (**A**) cells exposure to H_2_O_2_ (500 µmol/L, 60 min) in the presence of *Fucus spiralis* fractions (1000 µg/mL) and ascorbic acid (AA, 1000 µg/mL); (**B**) cells exposure to UVB (15 min), in the presence or absence of *Fucus spiralis* fractions (1000 µg/mL) and ascorbic acid (AA, 1000 µg/mL). Bars correspond to mean ± SEM of three independent experiments. Symbols (*) represent significant differences (One-way ANOVA, Dunnett’s test; *p* < 0.05) when compared to the vehicle.

The ability of *F. spiralis* fractions to decrease ROS production on HaCaT cells exposed to H_2_O_2_ and UVB radiation was studied, and the results are presented in Figure 6.

The pre-treatment with F2, F3, F4, F6, F7, F10, and F11 fractions drove to a significant decrease of ROS production in H_2_O_2_-stimulated HaCaT cells for the levels similar to the positive control, ascorbic acid (Figure 6A). On the other hand, in the presence of fractions F1, F5 and F8, ROS production was not affected. Regarding UVB-stimulated HaCaT cells (Figure 6B), it is possible to observe that the pre-treatment with F4, F6, F7, F10, and F11 fractions led to a significant decrease of ROS levels when compared to the vehicle. Among them, the highest protective effect was exhibited by fractions F7 and F10 on H_2_O_2_ assay, with an IC_50_ of 99.5 µg/mL (51.2–193.3) and 41.3 µg/mL (12.62–135.3), respectively. In the same way, fractions F7 and F10 exhibited the lowest IC_50_ on UVB assay with a value of 100.3 µg/mL (34.3–293.4) and 31.3 µg/mL (17.8–54.9), respectively.

### 3.6. UV–Visible Absorption Spectra

**Figure 7:**
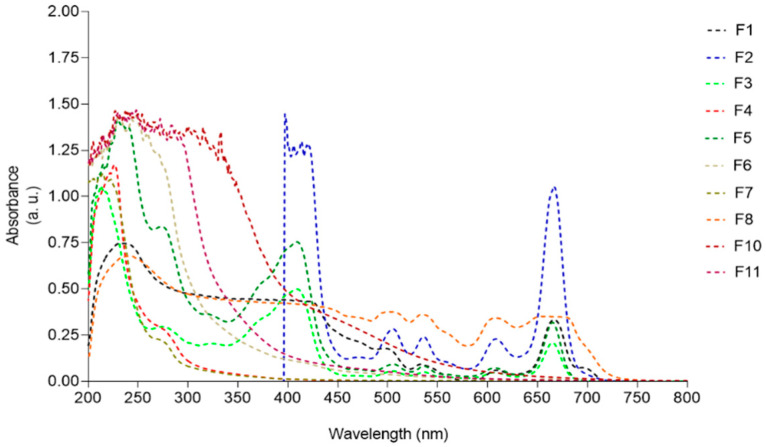
UV-visible absorption spectra (200–800 nm) of *Fucus spiralis* fractions.

In order to correlate the photoprotective capacity of *F. spiralis* fractions (F1-F11) with their chemical profile, the UV-visible spectrum of each sample was analyzed and the results are displayed in Figure 7.

Samples F1, F2, F3, F5, and F8, containing great amounts of pigments, presented absorption maxima peaks characteristic of chlorophylls (420–680 nm) and carotenoids (430–500 nm). Fractions containing more hydrophilic compounds such as F4, F6, F7, and F11 exhibited absorption peaks ranging from 210 to 280 nm. A broad UV absorption spectrum was exhibited by fraction F10, covering the UVA (320–400 nm), UVB (280–320 nm), and UVC (200–280 nm) ranges.

### 3.7. Chemical Caracterization by LC-MS

The chemical characterization of the most bioactive fraction from *F. spiralis* (F10) was performed through the analysis of LC-MS spectra in the negative and positive ion modes (Table 3). Tentative assignment was considered only if it was observed simultaneously the presence of peaks [M−H]^−^ and [M+H]^+^, confirming the phlorotannin MW. In addition, in some cases, it was observed the appearance of [2M+H]^+^ and [M−2H]^2−^ peaks [31], which can be regarded as an additional confirmation of the presence of the molecular species M. The obtained mass spectra were compared with literature data [32,33,34,35,36], allowing the tentative identification of the following compounds and/or their isomers: trifucol (*m*/*z* 373/375), tetrafucol (*m*/*z* 497/499), trifucophlorethol (*m*/*z* 621/623), hexafucol/difucotetraphloroethol/trifucotriphloethol (*m*/*z* 745/747), difucotetraphloroethol/trifucotriphloethol (*m*/*z* 869/871), and tetrafucotetraphlorethol/pentafucodiphlorethol/hexafucophlorethol (*m*/*z* 993/995).

## 4. Discussion

Three complementary in vitro assays were used to evaluate the antioxidant properties of *F. spiralis* fractions: their capacity to scavenge the free radical DPPH, the ability to reduce ferric iron (FRAP), and their oxygen radical absorbance capacity (ORAC). In addition, the determination of the total phenolic content (TPC) of each fraction, allowed to establish a relationship between the antioxidant potential and phenolic content of each sample. From the analysis of Table 1, it is evident that, in higher or lower degree, all fractions exhibited antioxidant properties at least in one or two antioxidant assays. However, fraction F10 revealed the strongest potential in the three in vitro assays which can be co-related with its highest content of phenolic compounds, namely phlorotannins. The antioxidant capacity of this group of compounds is fully reported in literature not only for several species of brown macroalgae but also for *Fucus* spp. [10,18,32,37,38,39]. However, when comparing results with those reported in literature, some reservations should be taken into account. Effectively, when working with natural biomass, several factors can influence final results, e.g., seaweed harvesting place, vegetative cycle, seasonal and climatic alterations, allied to different methodologies of extraction, fractionation, bioactivity and enzymatic assays, among others. An optimization of phlorotannins extraction from *Fucus vesiculosus* was recently reported [36] but no comparisons are made with those obtained herein, since different macroalgae species were studied and different solvents’ ratios were used. Besides the strong antioxidant potential of the phlorotannin enriched fraction F10, also the ethanolic fractions F3 and F5 exhibited significant antioxidant properties in all the performed in vitro assays, and will be soon subjected to more detailed studies regarding their chemical characterization.

The incorporation of natural antioxidants in dermo-cosmetic formulations is extremely important, since they protect skin cells from ROS which are involved in skin aging and skin cells deterioration. Besides cellular oxidative stress, with aging some of the main components of human skin e.g., collagen, elastin and hyaluronic acid decrease gradually leading to wrinkles and skin weakened structure mainly due to the degradation performed by the enzymes collagenase, elastase and hyaluronidase. Therefore, the research of new ingredients that can revert these signs is growing and marine environment has proven to be a good source of compounds with recognized efficacy in this field as fully reported [10,11,40,41,42,43].

A tentative correlation between the antioxidant and anti-enzymatic activities of the studied samples was performed through the principal component analysis (PCA) (Figure 2). This analysis evidenced that there is a clear correlation between the strong antioxidant potential of fraction F10 and their capacity to inhibit both collagenase and elastase. In turn, the inhibition over collagenase, elastase, and hyaluronidase promoted by fraction F4 seems to be correlated with other factors, since this fraction did not evidence high phenolic content nor high antioxidant activities, at least in the DPPH and FRAP assays. The strong antioxidant potential of F10, as well as its ability to inhibit collagenase and elastase can be attributed to its high concentration of phlorotannins. This group of compounds, isolated from different brown macroalgae, including *Fucus* species, are largely reported for their dermo-cosmetic potential due to its antioxidant, photoprotective, and anti-enzymatic properties [10,37,39]. The inhibition of hyaluronidase by a phlorotannin enriched extract obtained from *F. spiralis* was reported by Ferreres et al. [32] showing an IC_50_ of 730 µg/mL. Although in the present study it was not possible to determine the IC_50_ of F10 for the hyaluronidase inhibition, it is important to note that the methodologies used for extraction and to evaluate the anti-enzymatic inhibition are different, which can explain, at least in part, such results.

Another group of compounds largely reported for their anti-enzymatic properties are the sulfated polysaccharides [10]. Effectively, besides phenolics, it is expected that the most hydrophilic fractions F4, F7, and F11 retain high amounts of these macromolecules, including fucoidan, the sulfated polysaccharide commonly found in brown macroalgae. As reported by Kim et al. [44] numerous in vitro and in vivo studies showed that algal carbohydrates have various biological activities against skin disorders including hyperpigmentation, wrinkles, skin inflammation, and skin cancer. In particular, fucoidan suppressed mRNA and protein expression of matrix metalloproteinase MMP-1 upregulation, and type 1 pro-collagen downregulation stimulated by UVB, indicating that fucoidans present skin anti-aging potential with varied mechanisms of action.

It is well established that microbial diversity and relationships between members of cutaneous microbiota are essential for the maintenance of a healthy skin. The three microorganisms here assayed make part of this natural microbiome but are also associated with various skin disorders and bloodstream infections. *S. epidermidis* is a facultative anaerobic bacteria and, although it is not pathogenic for healthy people, it may represent a threat to patients with a compromised immunological system, and to those who need catheters or other surgical implants due to its capacity to form biofilms. Regarding *C. acnes*, it is predominantly found within follicles and pores, but it also lives in the surface of healthy skin. To survive, these bacteria use skin sebum and cellular metabolic by-products as nutrients. Contrary to what it was previously thought, acne vulgaris is not the result of a greater proliferation of all *C. acnes* strains, as patients with acne do not have more *C. acnes* in follicles than normal individuals [45]. Instead, acne might be triggered by the selection of a subset of *C. acnes* strains, including the acne-associated phylotype IA1, probably enhanced by a hyperseborrheic environment [45]. *Malassezia* yeasts can be considered as the almost exclusively single eukaryotic member of the microbial flora of the skin [46]. *M. furfur* as other *Malassezia* species occur on human skin as commensals, and they are associated with multiple skin disorders, such as pityriasis versicolor, folliculitis, seborrheic dermatitis/dandruff, atopic dermatitis, and psoriasis [47]. In a first approach, it seems that the growth inhibitory effect of fraction F1 over the assayed microorganisms can be attributed to the lipophilic compounds present in this sample, while the most hydrophilic fraction F7 revealed to be active against *M. furfur*. On the contrary, it was observed a growth stimulation of *C. acnes* by the hydrophilic fractions F7 and F11, probably due to their content in sugars, proteins and minerals. The growth stimulation of the enriched phlorotannin fraction F10 over *S. epidermidis* can be regarded as a positive contribution since recent data have shown that *S. epidermidis* and *C. acnes* interact together, and are critical in the regulation of skin homeostasis [45]. As reported by these authors, another way to maintain cutaneous health can be achieved by supplementing the skin microbiota with probiotics to shift the balance towards a healthy microbiome. Although phlorotannins are fully reported for their antimicrobial activities [48], including the inhibitory activity promoted by phlorotannins purified extracts from *F. spiralis* over *S. epidermidis* (MIC = 3.9 mg/mL), it seems that the concentration here used (1 mg/mL) is not effective against the studied microorganisms.

In vitro cell line assays are an alternative to animal testing and a valuable tool for a preliminary evaluation of natural or synthetic compounds efficacy and security for further dermatological applications. In this work, three different cell lines were used, the fibroblast cells from Swiss albino mouse embryo tissue (3T3), adult human skin keratinocytes (HaCaT), and mouse macrophage cells (RAW264.7). Once it was observed that at the maximum concentration (1000 µg/mL), all fractions (excepting F7) induced cytotoxicity on 3T3 cell line, in vitro assays with the most promising sample (F10) were performed at sub-toxic concentrations (300 µg/mL). Concerning HaCaT cells, only the most lipophilic fractions F1 and F8 induced significant cytotoxicity at 1000 µg/mL. Due to their weak bioactivity potential, no cytotoxicity assays were performed at lower concentrations.

Several macroalgae derived-components demonstrated to be effective in reducing inflammatory mediators such as NO, TNF-α, IL-6, and IL-1β, in downregulating inflammatory enzymes like iNOS and COX-2, and in modulating the signaling pathways that lead to NF-κB activation [49]. The anti-inflammatory potential of *F. spiralis* fractions was assessed through their capacity to inhibit the production of nitric oxide (NO) by lipopolysaccharide (LPS)-stimulated RAW 264.7 macrophage cells. Macrophages play an important role in inflammatory pathologies, being related with an overproduction of inflammatory mediators, including NO [48]. So, the research of novel and effective NO inhibitors is relevant for the prevention and treatment of inflammatory impairments associated with a broad range of diseases including several cutaneous pathologies. At sub-cytotoxic concentrations, fractions F1, F5, and F8, mainly constituted by apolar components, revealed to be the most effective in reducing NO production in the tested model. These results are in line with those reported by Lopes et al. [50] which investigated the glyceroglycolipids composition of *F. spiralis* collected off the Portuguese coast. The isolated lipophilic components, e.g., monogalactosyl diacylglycerols and a monoacylglycerol, displayed anti-inflammatory activity through the inhibition of NO release by macrophages. Seca et al. [51] also identified another acylglycerol, 1-palmitoylglycerol, highlighting the potential of *F. spiralis* as a source of bioactive acylglycerols. However, the ethanol fractions F3 and F6 also evidenced significant capacity to inhibit the NO production and are selected for further studies regarding their chemical characterization aiming the establishment of structure-bioactivity relationships. In addition, it is also interesting to observe that, at higher concentrations, almost all fractions did not promote NO production, meaning that they do not induce a spontaneous inflammatory status.

The production of reactive oxygen species (ROS) by HaCaT cells after exposure to H_2_O_2_ or UVB light was also evaluated. With the exception of fractions F1, F5 and F8, mainly constituted by lipophilic compounds, all the assayed fractions revealed a protective effect on HaCat cells submitted to H_2_O_2_ and UV oxidative stress. In particular, fractions containing high concentration of sulfated polysaccharides (F7) and phlorotannins (F10) evidenced a strong protective effect against ROS production promoted by H_2_O_2_ and UV radiation. The antioxidant and photoprotective effects of these group of compounds obtained from seaweeds in general, and from *Fucus* species in particular, were recently reviewed [10,37,44], supporting the results here obtained. Effectively, systematic exposure of human skin to UV radiation promotes severe skin injuries like sunburns, aging, inflammation, and photo-carcinogenesis [52]. As many other marine organisms, seaweeds are highly exposed to UV radiation and, as a consequence, they have developed a mechanism of defence to counteract or minimize radiation damage through the biosynthesis of photoprotective substances like pigments, mycosporine-like amino acids, sulfated polysaccharides, and phenolic compounds [10,19,44,52,53]. Among this last group, phlorotannins are known for their strong antioxidant and photoprotective properties. Up to a certain concentration, these compounds are not cytotoxic, highlighting their potential as a safe dermo-photoprotector. It is believed that the photoprotection promoted by phlorotannins is correlated with their radical scavenging activity, since the hydroxyl groups bounded to the aromatic rings act as an electron donor, giving it to free radicals or other reactive species. Moreover, the inhibition of ROS-mediated damage on macromolecules inhibits the activation of the signal transduction pathways like the mitogen-activated protein kinases (MAPK) [53].Photoprotective potential was also revealed by the hydrophilic fractions F4, F6, F7, and F11, probably due to the presence of other phenolics and fucoidan, the most abundant sulphated polysaccharide found in brown macroalgae, which is reported for its photoprotective activity [19,44]. According to Pangestuti et al. [53] there are evidences that the photoprotection of fucoidan is mediated through the suppression of matrix metalloproteinase-1 (MMP-1) activity, the major enzyme implicated in the collagen damage and photoaging of UV-irradiated human skin. Fucoidan has been used for transdermal formulations targeting inflammatory skin conditions, for the treatment of thrombosis, vascular permeability diseases, subcutaneous wounds, and burns [54]. These authors reported the pharmacokinetics of fucoidan after topical application to rats, and concluded that fucoidan in ointments penetrate the skin barrier and accumulate in the striated muscle. Some mechanisms of radical scavenging, and the anti-inflammatory, anti-hyaluronidase and anti-coagulant bioactivities of fucoidan from *Fucus vesiculosus* were also reported [55,56], suggesting that MW, sulphate and fucose content, and polyphenols may contribute to these activities. However, amongst the studied extracts, the phlorotannin-enriched fraction F10 revealed to be the most promising one since it displayed the best performance in the in vitro bioactivities here assayed. For this reason, it was selected for a more detailed chemical characterization by LC-MS. Phlorotannins are complex phloroglucinol-based compounds and, despite the use of powerful advanced analytical methods like nuclear magnetic resonance spectroscopy and tandem mass spectrometry, due to their high degree of polymerization, type of linkage, and number of additional hydroxyl groups, their isolation and chemical characterization continues to be a challenge [34,35,36]. More than polymerization degree, hydroxyl groups availability determines phlorotannin antioxidant capacity. This suggests that linear structured isomers are more effective when compared with similar branched phlorotannin isomers, which might fold in a way that encloses the OH groups inside the structure, diminishing their antioxidant properties [35,57]. This shows the importance of SAR studies as reported by Załuski et al. (2017) [58], which also found a high correlation between antioxidant and anti-hyaluronidase activity and TPC from *Eleutherococcus* spp. However, as reported by these authors, chemical interactions (synergism, antagonism, and additional effects) among phenolic and non-phenolic compounds may occur, which may influence extracts’ bioactivities.

## 5. Conclusions

In conclusion, among the *F. spiralis* samples here studied, the enriched phlorotannin fraction F10, due to its broad spectrum of bioactivities, proved to be suitable for further dermo-cosmetic applications through their inclusion in skin formulations with antioxidant, anti-enzymatic, and photoprotective properties, allied to its role in the maintenance of skin microbiota homeostasis. Meanwhile, additional research is now in development regarding the isolation, structural characterization, and SAR studies of compounds from the remaining *F. spiralis* extracts, in particular the fucoidan-rich fractions, which have revealed also a good potential for further dermatological applications.

## Figures and Tables

**Table 1:** Yields and antioxidant capacity of *Fucus spiralis* fractions.

Fraction	Yield (%)	TPC ^a^	DPPH ^b^	ORAC ^c^	FRAP ^d^
F1	1.43	62.7 ± 6.0	645.7 (588.3–708.6)	1319.6 ± 162.5	333.8 ± 47.1
F2	1.24	230.9 ± 11.2	235.1 (260.4–267.6)	3214.4 ± 237.9	700.9 ± 118.9
F3	10.31	362.1 ± 9.7	157.6 (113.1–219.8)	3557.9 ± 243.1	1821.8 ± 253.7
F4	1.94	48.6 ± 7.9	>1000	1755.9 ± 275.3	504.0 ± 56.3
F5	6.99	309.5 ± 12.7	244.7 (208.9–286.8)	4238.1 ± 134.6	1209.4 ± 114.4
F6	6.61	95.1 ± 7.3	>1000	2794.1 ± 269.1	505.2 ± 69.3
F7	0.73	272.0 ± 26.7	631.5 (522.0–764.0)	3518.7 ± 376.8	1602.1 ± 95.4
F8	1.83	197.7 ± 8.4	257.2 (231.4–285.8	3319.4 ± 138.3	674.3 ± 98.2
F9	0.01	-	-	-	-
F10	0.65	1679.8 ± 34.0	38.5 (29.51–50.36)	16464.5 ± 1223.4	2378.2 ± 93.5
F11	11.34	121.5 ± 4.8	324.5 (289.4–363.8)	2353.6 ± 88.9	735.1 ± 102.7
BHT	-	-	164.5 (142.7–189.7)	142.9 ± 9.1	2821.5 ± 51.5

^a^ phloroglucinol equivalents/extract (mg PE/g); ^b^ radical scavenging activity (EC_50_ µg/mL); ^c^ Trolox equivalents/extract (µmol TE/g); ^d^ µmol/L FeSO_4_/g extract. EC_50_ values were determined for a 95% confidence interval.

**Table 2:** Enzymatic inhibitory activities (IC_50_, µg/mL) of *Fucus spiralis* fractions.

Fraction	Collagenase	Elastase	Hyaluronidase
F1	>1000	>1000	148.9 (122.2–181.3)
F2	>1000	>1000	79.5 (66.1–95.6)
F3	512.9 (442.8–594.1)	>1000	348.8 (290.5–418.7)
F4	40.4 (26.2–62.5)	67.8 (47.6–96.6)	61.1 (47.3–78.9)
F5	89.9 (69.1–117.1)	409.0 (293.9–569.2)	>1000
F6	156.7 (123.6–198.5)	>1000	128.8 (103.0–161.1)
F7	4.3 (3.5–5.3)	123.8 (103.0–148.7)	>1000
F8	391.6 (328.7–466.6)	>1000	>1000
F10	0.037 (0.009–0.142)	3.0 (2.5–3.6)	>1000
F11	31.3 (28.4–34.5)	586.5 (445.8–771.6)	110.1 (89.1–136.1)
EGCG	4.8 (4.1–5.5)	113.9 (80.7–160.0)	119.1 (126.1–320.4)

**Table 3:** Tentative identification of phlorotannins present in the ethyl acetate fraction (F10) of *Fucus spiralis*.

M (*m*/*z*)	[M−H]^−^ (*m*/*z*)	[M+H]^+^ (*m*/*z*)	Other Ions	Tentative Assignment	References
374	373	375	-	Trifucol	[34,35,36]
498	497	499	-	Tetrafucol or Fucodiphloroethol or their isomers	[32,33,35,36]
498	497	499	-		
622	621	623	-	Trifucophlorethol or its isomers	[34,35,36]
746	745	747	1493 [2M+H]^+^	Hexafucol or its isomers	[31,32,33,34,35,36]
746	745	747	1493 [2M+H]^+^		
870	869	871	-	Difucotetraphloroethol or Trifucotriphloethol or their isomers	[32,33,35,36]
870	869	871	-		
994	993	995	496 [M−2H]^2−^	Tetrafucotetraphlorethol, or Pentafucodiphlorethol or Hexafucophlorethol or their isomers	[32,33,36]
994	993	995	496 [M−2H]^2−^		
994	993	995	-		
